# Postoperative Analgesic Effects of Laserpuncture and Meloxicam in Bitches Submitted to Ovariohysterectomy

**DOI:** 10.3390/vetsci7030094

**Published:** 2020-07-21

**Authors:** Rubia M. Tomacheuski, Marilda O. Taffarel, Guilherme S. Cardoso, Ana A. P. Derussi, Marcos Ferrante, Rodrigo Volpato, Stelio P. L. Luna

**Affiliations:** 1 Department of Anesthesiology, Medical School, São Paulo State University (Unesp), Botucatu 18618687, Brazil; rubia.mitalli@unesp.com; 2 Department of Veterinary Medicine, Maringá State University (UEM), Umuarama 87502970, Brazil; motaffarel@uem.br; 3 Department of Veterinary Clinics, Londrina State University (UEL), Londrina 86057970, Brazil; vetschiess@gmail.com; 4 Department of Animal Reproduction, José do Rosário Vellano University (UNIFENAS), Alfenas 37131587, Brazil; ana_pagnano@yahoo.com.br; 5 Department of Veterinary Medicine, Federal University of Lavras (UFLA), Lavras 37200900, Brazil; marcos.ferrante@ufla.br; 6 Department of Veterinary Surgery and Animal Reproduction, School of Veterinary Medicine and Animal Science, São Paulo State University (Unesp), Botucatu 18618000, Brazil; rodrigo.volpato@hotmail.com

**Keywords:** pain, dogs, low-level laser therapy, acupuncture, analgesia

## Abstract

Background: This prospective, randomised and blind study investigated the efficacy of laserpuncture for postoperative pain management in dogs. Method: Sixteen bitches were sedated with acepromazine and randomly treated before ovariohysterectomy with meloxicam 0.2 mg·kg^−1^ intramuscular or laserpuncture (wavelength 904 mm, frequency 124 Hz, potency 10 Joules, 100 s in each acupoint). Anaesthesia was performed with propofol, isoflurane/O_2_, and fentanyl. The Glasgow Composite Measure Pain Scale (GCMPS) and Dynamic Interactive Visual Analog Scale (DIVAS) were used to evaluate postoperative pain before and for 24 h after surgery. Morphine was administrated as rescue analgesia when pain scores were ≥3.33 (GCMPS). Differences between treatments, time points, and amount of rescue analgesia between groups were investigated by the Mann–Whitney test and the area under the curve (AUC) for GCMPS, Friedman, and Chi-squared tests, respectively (*p* < 0.05). Results: Dogs treated with laserpuncture presented lower GCMPS AUC for 24 h and lower GCMPS scores at 2 and 4 h postoperatively (*p* = 0.04). Three dogs treated with meloxicam required postoperatively rescue analgesia against none treated with laserpuncture. Conclusions: In this preliminary study, laserpuncture mitigated postoperative pain in dogs following ovariohysterectomy, and the technique is a promising adjunct to perioperative pain management in dogs undergoing soft tissue surgery.

## 1. Introduction

Preemptive and multimodal preoperative analgesia prevents the occurrence of allodynia and hyperalgesia in the postoperative period [1]. Nonsteroidal anti-inflammatory drugs (NSAIDs) are among the most used analgesics to control postoperative pain, however, they may cause considerable adverse effects, such as gastrointestinal disorders, and are contraindicated in dogs with renal disease and coagulation disorders [2,3]. Meloxicam is a popular NSAID and commonly reported to treat postoperative pain in dogs subjected to ovariohysterectomy [4].

Acupuncture can be used to treat perioperative pain in dogs [5]. The most recent Pain Management Guidelines for Dogs and Cats from the American Animal Hospital Association affirmed that acupuncture should be strongly considered as a nonpharmacological modality for multimodal pain management [1].

Various techniques may be used to replace or complement the effect of dry needle acupuncture. The main techniques are electroacupuncture [6], pharmacopuncture [7], and laserpuncture [8]. Laserpuncture consists of using a light amplification by stimulated emission of radiation (LASER) to stimulate acupoints. Its use and mechanisms of action have been described previously [9]. LASER is a noninvasive, painless procedure that may be used to stimulate acupoints and is especially indicated for animals that do not tolerate needles. LASER presents local anti-inflammatory effects in the affected area [10]. In cats undergoing ovariohysterectomy, laserpuncture reduced postoperative analgesic requirements [8,11].

The choice of adequate acupoints is important for an effective analgesic effect for any acupuncture technique. Electroacupuncture performed in Large Intestine 4 and Stomach 36 acupoints, either used in isolation or combined with other points, abated visceral pain in humans [12] and postoperative pain in cats [10] and dogs [13].

Pain assessment is a challenge in animals. Pain scales improve the repeatability and reproducibility of evaluations and minimize the evaluator’s influence [14,15]. GCMPS was validated for acute pain assessment. It is a scoring questionnaire based on changes in spontaneous and interactive behaviour [16].

Considering that laserpuncture might be an alternative to NSAID to prevent postoperative pain, this study aimed to investigate the effect of laserpuncture versus meloxicam for postoperative pain management in bitches submitted to ovariohysterectomy. The hypothesis was that laserpuncture provides effective postoperative analgesia.

## 2. Materials and Methods

The School of Veterinary Medicine and Animal Science (University of São Paulo State—UNESP) Ethical Committee for the Use of Animals in Research approved this study (protocol 180/2010). Owners of all the animals used signed an informed consent term.

### 2.1. Animals

Twelve crossbred, one Sharpei, one Labrador, and two Poodle bitches (*n* = 16) were used. Inclusion criteria were: clinically healthy dogs following clinical examinations and blood tests (haematology) [17]. The study was opportunistic and selected animals for a reproduction study. Most of the dogs were in the final phase of oestrus and had been submitted to copulation to produce embryos. Exclusion criteria were patients aged less than six months or more than ten years, and any abnormality observed on clinical examination or blood tests.

### 2.2. Procedures

On the day before the surgery, the dogs were admitted and allocated in individual kennels, appropriate to the species and size of the animal, where they stayed during the whole period of evaluation. After water fasting of two hours and food restriction of eight hours, all dogs received acepromazine (0.05 mg·kg^−1^; Acepran 0.2%; Vetnil, Louveira, SP, Brazil) via the intramuscular route (IM). Ten minutes later, after randomization performed by drawing, dogs were allocated into two groups of equal numbers: meloxicam (MG; *n* = 8) or laserpuncture (LG; *n* = 8). The MG dogs received meloxicam IM (0.2 mg·kg^−1^; Maxicam 0.2%; Ourofino Saúde Animal, Cravinhos, SP, Brazil). The LG dogs were submitted to bilateral laserpuncture. The sequence of the acupoint stimulation was from thoracic to pelvic limbs, from distal to proximal regions, according to the following order: Large intestine 4 (LI4), Liver 3 (Liv3), Spleen 6 (Sp6), Stomach 36 (St36), and Gallbladder 34 (GB34). Anatomical location is described elsewhere [18]. The laserpuncture was provided by an infra-red LASER (Laservet, São José dos Campos, SP, Brazil), wavelength 904 mm, frequency of 124 Hz, and potency of 10 Joules. Each acupoint was stimulated for 100 s. Total treatment time ranged between 17 and 20 min. The regions of stimulation were not shaved; however, the handler deviated the dog’s hair with her hands to perform laserpuncture. The potency was always the same, regardless of skin colouration.

Propofol (Propovan, Cristália, São Paulo, SP, Brazil), in a sufficient dose (up to 5 mg·kg^−1^ IV) to cause the effective loss of the laryngotracheal reflex [19], was administered intravenously (IV) 40 min after acepromazine. Anaesthetic maintenance was performed with isoflurane (Isoforine, Cristália, São Paulo, SP, Brazil) vaporised in oxygen (O2), using a rebreathing circuit. The vaporiser setting was adjusted to maintain a surgical-anaesthetic plane based on the eye position, mandibular tonus, and absence of autonomic reflex to nociceptive stimulus. Vaporiser setting was increased when heart rate or arterial blood pressure was greater than 20% compared to pre-surgical values. Fentanyl (2.5 µg·kg^−1^ IV; Fentanest; Cristália, São Paulo, SP, Brazil) was administered just before the beginning of surgery. Lactated ringer solution (10 mL·kg^−1^·h^−1^; Fresenius Kabi Brasil AS, Aquiraz, CE, Brazil) was administered IV during surgery.

The same experienced surgeon (R.V.) performed all surgeries. In all cases, the surgery consisted of median laparotomy followed by exposition and resection of ovaries, uterus resection, and suture. Intraoperative monitoring was performed with a multiparametric monitor (LifeWindowTM Lite, Digicare Animal Health, Rio de Janeiro, RJ, Brazil). Measurements included pulse oximetry, heart and respiratory rates, and ETCO_2_; the rectal temperature was monitored with a digital thermometer (Becton Dickinson, São Paulo, SP, Brazil) and systolic arterial blood pressure using a Doppler (811-B Ultrasonic Doppler Flow Detector, Parks Medical Electronics, INC., Aloha, OR, USA).

### 2.3. Outcome Measures

Pre- and postoperative pain were assessed by the same evaluator (G.S.C.), blinded to the treatment, always following the same sequence: GCMPS [16,20], followed by evaluation of sedation by the Simple Descriptive Scale (SDSsed)—where: 0 = fully alert and able to stand and walk; 1 = alert, able to maintain sternal recumbency and walk but might be ataxic; 2 = drowsy, able to maintain sternal recumbency but unable to stand; and 3 = fast asleep, unable to raise head [16] and DIVAS [21]. DIVAS was a Visual Analog Scale combined to a dynamic and interactive component, based on approach and interaction with the animal, and palpation of the wound. The evaluator scored the degree of pain in a 100 mm line, where 0 corresponded to no pain and 100 to the worst possible pain [21]. For pain assessment, the evaluator first observed the dog in the kennel, then opened the kennel, called the animal, encouraged the dog to walk, and palpated the area around the wound [16].

The pain and sedation evaluations were performed on the day before surgery (T-24), immediately before the procedure (T0), and 1, 2, 4, 6, 8, 12, and 24 h after extubation (T1 to T24). The rescue analgesia consisted of morphine (0.5 mg·kg^−1^ IM) and was administered when the pain score, based on GCMPS, was ≥3.33 points, equivalent to 1/3 of the scale total value and corresponding to moderate pain, according to a previous study [22].

### 2.4. Statistical Analysis

Data normality was tested by the Shapiro–Wilk test. As data did not present normal distribution, the Mann–Whitney test was used to compare differences between treatments, the Friedman Test to compare differences for time point in each group. Corrections for multiple comparisons were performed for both groups. Furthermore, to compare treatments, differences in AUC of the GCMPS scores were calculated. The Chi-square test was employed to compare the number of rescue analgesia administered between groups. An unpaired *t*-test compared differences in age and weight between groups. A *p*-value < 0.05 indicated significant differences. To determine the sample size before the study, it was deemed that a difference of 1 point (standard deviation 0.7) in the mean GCMPS scores, considering a test power of 80% and alpha of 0.05. Data were analysed using BioEstat 5.3 (free software, Instituto de Desenvolvimento Sustentável Mamirauá, Brazil).

## 3. Results

Dogs of the laserpuncture and meloxicam group weighed 11.2 ± 6.6 kg (5–21), and 11.2 ± 10.5 kg (3.4–36) kg and were 33 ± 11 months old (12–108), and 49 ± 11 months old (7–96), respectively. The propofol dose used to induce anaesthesia was 5 ± 0.1 and 4.4 ± 1.8 mg·kg^−1^ in dogs treated with laserpuncture and meloxicam, respectively. The surgery lasted 15.25 ± 4.8 min for the laserpuncture group and 15.25 ± 6.5 min for the meloxicam group. There were no differences in weight (*p* = 0.674), age (*p* = 0.172), dose of propofol (*p* = 0.377), and surgical time (*p* = 1) between groups.

**Figure 1:**
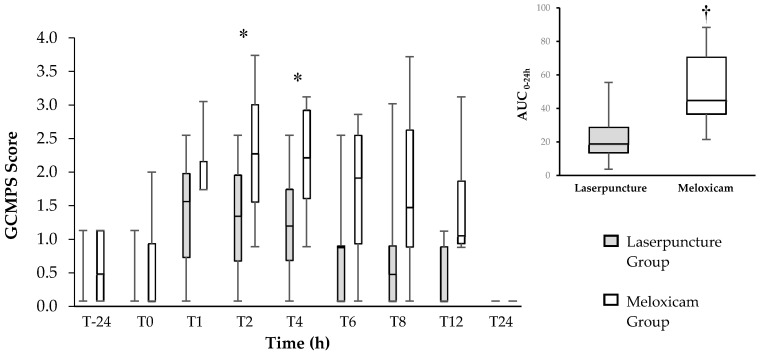
**Left**—Box plot of the Glasgow Composite Measure Pain Scale (GCMPS) scores over time (center horizontal lines express the medians; box limits indicate the 25th and 75th percentiles, whiskers extend 1.5 times the interquartile range from the 25th and 75th percentiles) for 16 bitches treated preoperatively with laserpuncture (*n* = 8) or meloxicam (*n* = 8) and submitted to ovariohysterectomy. * Indicates the differences between groups at T2 (*p* = 0.042) and T4 (*p* = 0.022) according to Mann–Whitney test. **Right**—Box plot of the area under the curve (AUC) of the GCMPS scores (center horizontal lines express the medians; box limits indicate the 25th and 75th percentiles, whiskers extend 10th and 90th percentiles) between 0–24 h. † Indicates difference between groups (*p* = 0.007).

**Figure 2:**
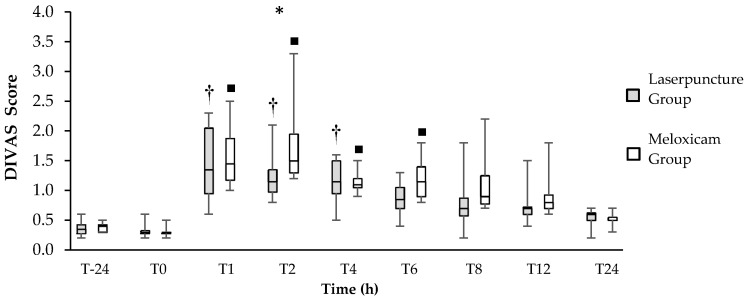
Box plot of the Dynamic Interactive Visual Analogue Scale (DIVAS) scores over time (center horizontal lines express the medians; box limits indicate the 25th and 75th percentiles, whiskers extend 10th and 90th percentiles) for 16 bitches treated preoperatively with laserpuncture (*n* = 8) or meloxicam (*n* = 8) and submitted to ovariohysterectomy. * Indicates the differences between groups at T2 (*p* = 0.020) according to the Mann–Whitney test. † Indicates differences between postoperative time points and T-24 and/or T0 in the Laserpuncture Group (*p* < 0.05). ■ Indicates differences between postoperative time points and T-24 and/or T0 in the Meloxicam Group (*p* < 0.05).

Dogs treated with laserpuncture demonstrated lower DIVAS and GCMPS scores than those treated with meloxicam at 2 (*p* = 0.04) for both scales, and 4 (*p* = 0.04) hours postoperatively for GCMPS (Figure 1; Figure 2). The area under the curve of the GCMPS scores in dogs treated with laserpuncture was significantly lower than in those treated with meloxicam (*p* = 0.0156) (Figure 1).

In the Friedman´s test comparison over time, there was no difference in the GCMPS scores (*p* = 0.12) or the DIVAS (*p* = 0.1211) before and after surgery in any of the treatments (Figure 1 and Figure 2). The DIVAS scores increased between T1 and T4 in dogs treated with laserpuncture when compared to T-24 and T0. In dogs treated with meloxicam, DIVAS scores were higher between T1 and T6, compared to T-24 and T0 (Figure 2). Based on the multiple comparison correction GCMPS scores increased in T4 and T12 compared to basal values, but there was no difference for DIVAS.

Although there was no significant difference, the dogs treated with meloxicam required more administrations of rescue analgesia, in a total of three animals (one animal at T2, another at T8, and one before T1 and also at T2), than dogs treated with laserpuncture, none of which required rescue analgesia.

There were no significant differences over time or between groups for SDSsed.

One dog treated with meloxicam vomited in the postoperative period (before T1). One dog treated with laserpuncture vomited 20 min after the end of surgery and had three subsequent events of vomiting in the postoperative period (T2, T8, and T12).

## 4. Discussion

Laserpuncture showed promising analgesic results; postoperative pain scores in dogs submitted to laserpuncture were lower than dogs treated with meloxicam up to 4 h after surgery and no rescue analgesia was required for 24 h. This study suggests that laserpuncture was at least as effective as meloxicam for treating postoperative pain or slightly better than meloxicam in the first four hours after surgery.

The increase in plasma β-endorphin concentration after acupuncture lasts between one and three hours in dogs [13]. This is usually the period when postoperative analgesic intervention after ovariohysterectomy is most required [22]. As rescue analgesia was not necessary for any of the dogs treated with laserpuncture; it seems that analgesia might be partially mediated by an increased β-endorphin concentration during this period. The dogs treated with meloxicam required intervention analgesia, therefore a close assessment of pain should be considered when only meloxicam is used for postoperative pain. In contrast to our results, in another study from our group using a similar methodology, meloxicam provided sufficient postoperative analgesia in dogs undergoing ovariohysterectomy, as none of the dogs required analgesic rescue [23]. The finding that three of eight dogs required analgesic rescue in the present study might be explained by the fact that the surgeon and the pain evaluator were different from the previous study. Although this was an opportunistic study, with an apparent small number of animals, it was possible to demonstrate a difference between treatments. Sample size calculation guaranteed that the number of animals was sufficient to avoid the type II error and the evidence of a statistical difference in pain scores indicates the beneficial effect of laserpuncture.

Postoperative pain has a strong inflammatory component, and the efficacy of NSAIDs to control postoperative pain in dogs submitted to ovariohysterectomy has been demonstrated in other studies [24,25], including effects superior to some opioids [26]; however, they can cause relevant adverse effects, such as gastrointestinal, liver, and kidney disorders [3,27]. Meloxicam is often used in veterinary medicine and recommended to prevent and treat surgical acute pain and musculoskeletal pain in dogs [1].

Central and peripheral sensitization is the main cause of pain in the first hours after surgical incision [28]. The mechanisms of acupuncture-induced analgesia involve activation of the opioidergic and serotonergic systems [29] and α2 spinal receptors [30], modulation of the expression of N-methyl-D-aspartate receptors [26], and modulation of endogenous opioids [31], such as endorphins, enkephalins, and dynorphins [32], thus contributing to inhibition of central and peripheral sensitization. The mechanisms behind the effect of laserpuncture are probably similar to dry needle acupuncture. It is suggested that laserpuncture stimulates specific brain areas, similar to those of the use of metal needles [25]. Low-intensity laser therapy affects the mitochondrial cytochromes, which absorb the red and near-infrared light [26]. This absorption increases ATP synthesis, the concentration of ‘reactive oxygen species’ [27], and nitric oxide [28]. Therefore, the analgesic effect was probably systemically mediated. In rats, laserpuncture at the ST36 acupoint induced antinociception, mediated by activation of the opioidergic and serotonergic systems [29]. In cats undergoing ovariohysterectomy, bilateral laserpuncture at Stomach 36 and Spleen 6 reduced the postoperative analgesic requirements compared to the control group, which received no acupuncture [8]. In dogs, acupuncture performed at the same acupoints use here, abated pain after ovariohysterectomy as effectively as NSAIDs and morphine [7]. In two studies in cats undergoing ovariohysterectomy, laserpuncture, using the same settings, except for 3 J.cm-2, for 9 s at Stomach 36 and Spleen 6 acupoints, reduced postoperative analgesic requirements [8,10]. Therefore, according to these previous studies and the present one, these settings seem appropriate to provide postoperative analgesia both in dogs and cats.

The absence of a gold standard tool for pain assessment in dogs is a limitation of the study. The scale used is the only interval scale with descriptors for dogs, and the attendance of descriptors on a scale minimizes the bias of the evaluator’s interpretation [33]. GCMPS was elaborated according to psychometric principles and subsequent construct, content, and sensitivity validation [20]. This scale was posteriorly evaluated by Murrel et al. [16]. They showed that the scale was able to distinguish different pain levels, even when the evaluator was not a native English speaker, as in our study. GCMPS was compared with other scales such as DIVAS, NRS, and the University of Colorado scale; GCMPS was more sensitive to determine the requirement for rescue analgesia and showed a strong correlation between both experienced and not experienced evaluators [34]. Another recent study comparing intra- and inter-rater agreement for DIVAS, NRS, and the Glasgow pain scale [20] has shown that for experienced evaluators, this scale has good and very good reliability [33]. However, a limitation of GCMPS is the absence of a defined cut-off point for analgesic intervention, whereas other studies have used values of 30 [35] and 35% [23] of the total score. In our study, the cut-off point corresponded to 1/3 of the total value (3.33), considered moderate pain.

The relatively low postoperative pain scores both in DIVAS and GCMPS may be explained by the use of preventive analgesics [36] and minimal surgical manipulation performed by an experienced surgeon [37]. The difference of findings in the evaluation between the scales used can be explained by the absence of descriptors in the DIVAS, which allows, as demonstrated by Hofmeister and others [33], that an experienced evaluator considers other factors than those established by the scale to determine the patient’s pain score.

The state of sedation may inflate the pain scores due to lack of response to stimuli and due to immobility. Sedation did not appear to affect our results because there was no difference along the time in each group or between the groups. Although pain assessment was performed before sedation assessment and, therefore, might have affected the sedation scores, in practical terms both evaluations were assessed together, still, only the forms were filled separately and following the same sequence. For pain assessment, the only contact performed with the dog was slight palpation of the surgical area. Considering that some sort of stimuli is required to assess sedation, this interference was not relevant as the same procedure was performed in both groups. Another point to be considered was that pain, and not sedation, was our primary outcome. The same applies to the two pain assessment methods. GCMPS assessment requires interaction, and so does DIVAS. Both tools have also been used previously to assess pain in a similar model [23].

Postoperative nausea and vomiting (PONV) are common adverse effects in dogs and must be prevented [38]. In this study, one dog from the meloxicam group vomited once, and one from the laserpuncture group vomited four times. Preoperative pharmacological antiemetic prophylaxis should be considered. Another alternative would be the use of the acupoint PC6, which diminished PONV in dogs [39].

A possible limitation of the study could be the administration of meloxicam IM. Meloxicam achieves 90% maximum plasma concentration within 30–50 min after IM injection in man [40], and the plasma concentration is analogous for humans and dogs [41]. In this study, the period between the meloxicam administration and the first pain assessment was longer than one hour, allowing sufficient time for absorption and biodisponibility [24].

## 5. Conclusions

In this preliminary study, laserpuncture mitigated postoperative pain in dogs following ovariohysterectomy, and the technique is a promising adjunct to perioperative pain management in dogs undergoing soft tissue surgery.
